# Preparation and the Photoelectric Properties of ZnO-SiO_2_ Films with a Sol–Gel Method Combined with Spin-Coating

**DOI:** 10.3390/s24237751

**Published:** 2024-12-04

**Authors:** Ziyang Zhou, Weiye Yang, Hongyan Peng, Shihua Zhao

**Affiliations:** 1 College of Physics and Electronic Engineering, Hainan Normal University, Haikou 571158, China; 2 The Innovation Platform for Academicians of Hainan Province, Haikou 571158, China

**Keywords:** ZnO-SiO_2_ composite films, sol–gel method, photoelectric performance, photoluminescence (PL), photoresponse, photoconductivity

## Abstract

This study explores the fabrication of ZnO-SiO_2_ composite films on silicon substrates via a sol–gel method combined with spin-coating, followed by annealing at various temperatures. The research aims to enhance the UV emission and photoelectric properties of the films. XRD showed that the prepared ZnO sample has a hexagonal structure. SEM images revealed the formation of ZnO nanorods within a dense SiO_2_ substrate, ranging from 10 μm to 30 μm in length. Photoluminescent analysis showed that the film exhibited strong UV emission centered at 360 nm. The response time measurements indicated that the optimal photoresponse time was approximately 2.9 s. These results suggest the potential of ZnO-SiO_2_ films as efficient UV-emitting materials with high photoconductivity and a stably reproducible response under visible light, thereby laying the foundation for their application in advanced optoelectronic devices.

## 1. Introduction

Zinc oxide (ZnO) has attracted widespread attention due to its exceptional properties, such as a high electron mobility, a wide direct bandgap (3.37 eV), and a relatively high exciton binding energy (60 meV) [1]. It has been extensively applied in gas sensors [2], photodetectors [3], photoluminescent devices [4], and composite catalysts [5]. Additionally, ZnO exhibits a variety of nanostructures, such as microspheres, nanorods, nanowires, nanorods, and flower-like structures. By precisely controlling the morphology and size of these nanostructures, ZnO has become a promising nanocomposite material system with broad application prospects.

In recent years, the growth of ZnO nanostructures on silicon (Si) substrates has garnered widespread attention due to their potential to enhance photodetection and photoluminescence performance. For instance, Ameer successfully fabricated single-crystalline ZnO nanorod arrays on silicon substrates using a wet chemical method, demonstrating excellent ultraviolet photocatalytic performance [6]. Althumairi et al. successfully prepared europium-doped ZnO microspheres on p-type Si substrates using a chemical bath deposition method and achieved tunable color emission by adjusting the Eu doping concentration and annealing temperature [7]. Hu significantly enhanced the electrical and optoelectronic properties of ZnO by preparing Cu-Al co-doped ZnO nanorods (NRs) thin films on Si substrates using both hydrothermal and sol–gel methods [8]. Moreover, Li employed plasma-assisted pulsed laser deposition (PLD) and hydrothermal methods to fabricate ZnO nanorod arrays and found that the ultraviolet emission intensity of ZnO was dependent on the type of contact at the metal/ZnO interface after metal modification of the ZnO surface [9]. These studies largely focus on ZnO nanowires (NWs) and nanorods due to their high surface-to-volume ratio. However, the surfaces of ZnO nanowires and nanorods often exhibit a high density of defects, such as oxygen vacancies, which can lead to both radiative and non-radiative recombination. To improve ultraviolet emission efficiency, these surface defects can be passivated and modified using materials such as SiO_2_ [10]. Due to the ultraviolet light sensitivity of ZnO nanorods with a high surface-to-volume ratio largely depending on their defects, if ZnO nanorods are coated with SiO_2_, their photoelectric response performance may induce significant changes.

Therefore, this study adopted a low-cost and simple sol–gel method combined with spin-coating to successfully fabricate ZnO-SiO_2_ thin films on silicon substrates via a one-step method. The prepared ZnO-SiO_2_ thin films not only exhibited strong ultraviolet luminescence but also demonstrated relatively fast photoelectric response under visible light irradiation. These results provide valuable insights for future research in related optoelectronic devices fields.

## 2. Materials and Methods

Zinc acetate dihydrate (ZAD), anhydrous ethanol (EtOH), tetraethoxysilane (TEOS), and cetyltrimethylammonium bromide (CTAB) were selected from Shanghai Macklin Biochemical Co., Ltd. (Shanghai, China). The hydrochloric acid (HCl) had a concentration of 37% and was obtained from Sinopharm Chemical Reagent Co., Ltd. (Shanghai, China). The silicon substrates were 15 mm squares of p-type (100) monocrystalline silicon with a resistivity of 10–20 Ω·cm. The water used was deionized water prepared from a pure water system, and all chemical reagents were of analytical grade and required no further purification before use.

The ZnO-SiO_2_ films were fabricated on silicon substrates using a sol–gel method combined with spin-coating. The fabrication process involved the following steps. Half of the EtOH was mixed with 3 mL of TEOS and stirred to form solution A. Simultaneously, the remaining EtOH, water, and HCl were mixed and stirred to form solution B. Solution A was slowly dripped into solution B over a 30 min period to form a uniform and stable solution C. The stirring of solution C continued for another 30 min, and then 0.263 g of CTAB and 0.400 g of ZAD were added into solution C, which was fully stirred for an additional 30 min to form the final mixed solution. The mixed solution was then allowed to age at room temperature for 24 h to form a sol. The volume ratio of TEOS, EtOH, H_2_O, and HCl was 3:20:1:1, respectively.

Before spin-coating, the silicon substrates were treated using an ultrasonic cleaner, repeatedly washed with alcohol and diluted hydrochloric acid, then rinsed with deionized water, and air-dried naturally. The spin-coating process was conducted at a fixed speed of 2000 rpm, with each spin lasting 30 s. After each layer was applied, the sample was dried in an oven at 70 °C for 20 min. This step was repeated four times. Finally, the samples were annealed in a tube furnace at 300 °C, 500 °C, and 700 °C for 1 h, respectively.

The fabricated films were characterized using various techniques. First, X-ray diffraction (XRD) (Rigaku Ultima-IV) with Cu Kα radiation (wavelength λ = 1.5406 Å) was used to determine the crystalline structure of the films over a 2θ range of 30° to 70°. Second, a JEOL JSM-7100F scanning electron microscope was used to observe the surface morphology. Raman spectra were measured using a RENISHAW Raman spectrometer with a 532 nm laser over the range of 100–1300 cm^−1^. Photoluminescence (PL) spectra were measured at room temperature using a Hitachi F-2600 fluorescence spectrometer, with a xenon lamp as the excitation source. Additionally, the photoelectric performance of the films was measured using a CHI660e electrochemical workstation and a DM4 probe test station. During testing, copper probes were spaced 5 mm apart, and a 5 W ring-shaped white LED light source was placed 25 cm from the film. In this experiment, the light power density irradiated onto the film was approximately 0.012 W/cm^2^. This configuration allowed for precise measurement of the photocurrent of the films under different lighting conditions, ensuring experimental consistency and reproducibility.

## 3. Results

### 3.1. XRD Analysis

**Figure 1:**
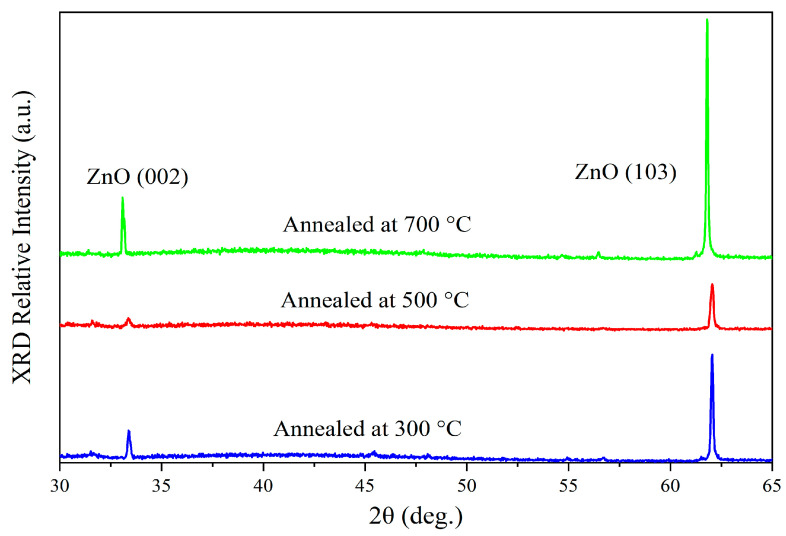
XRD patterns of the prepared samples annealed at 300 °C, 500 °C, and 700 °C.

Figure 1 is the X-ray diffraction (XRD) spectra of the thin film samples annealed at different temperatures, which presented that two peaks observed at 33.38° and 62.06° correspond to the (002) and (103) crystal planes of ZnO, respectively. The two diffraction peaks suggested that the samples have a wurtzite hexagonal structure of zinc oxide (JCPDS No. 36-1451) [11]. Moreover, these two peaks are shifted approximately 1° towards lower angles compared to the standard peaks, which may be attributed to the presence of compressive strain within the samples [12]. Additionally, the broadening of the diffraction peaks in the XRD spectra further indicates the existence of compressive strain in the samples [13].

The intensity of the XRD diffraction peaks shows different trends at annealing temperatures of 300 °C, 500 °C, and 700 °C, with the peak intensity at 500 °C being lower than that at 300 °C and 700 °C. This phenomenon can be explained in terms of the grain defects, interface stress, and the competition during grain growth.

At the annealing temperature of 300 °C, compared to the samples annealed at 700 °C, the lower temperature does not provide sufficient thermal energy for ZnO grains to grow and merge effectively. As a result, the crystallinity of the film remains relatively poor, leading to less pronounced grain development. This incomplete crystallization results in the broader and lower peaks in the XRD pattern.

At 500 °C, the ZnO crystals undergo rapid grain growth, with the grains enlarging and merging. However, the temperature is not high enough to fully eliminate the stress and defects at the grain boundaries. This leads to increased defect density, suppressing the intensity of some XRD peaks. It is suggested that the competition in grain growth and the introduction of interface defects are the main reasons for the lower peak intensity at 500 °C compared to 300 °C and 700 °C.

As the temperature increases to 700 °C, high-temperature annealing provides enough energy to repair defects and release interface stress, further promoting grain growth. With larger grains and improved crystallinity, the XRD peaks become sharper and stronger, reflecting the optimal enhancement in crystal quality.

### 3.2. SEM Analysis

**Figure 2:**
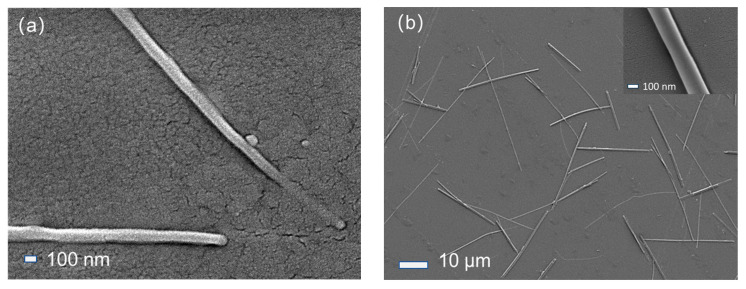
SEM images of ZnO-SiO_2_ films annealed at different temperatures: (**a**) annealed at 300 °C; (**b**) annealed at 500 °C.

Figure 2 presents the SEM images of ZnO-SiO_2_ films annealed at different temperatures. From Figure 2a, it is clear that the surface of the ZnO-SiO_2_ composite film is covered with a dense granular SiO_2_ structure, along with a small number of ZnO nanorods with diameters of approximately 100 nm and lengths of several micrometers. These ZnO nanorods are relatively short and sparsely distributed, suggesting that the lower annealing temperature limits the growth of ZnO nanostructures. Some of ZnO nanorods extend out from the SiO_2_ substrates, indicating that ZnO may begin growing within the SiO_2_ substrates and gradually extend outward, eventually penetrating the surface to form nanorods.

Figure 2b shows the SEM image of the ZnO-SiO_2_ composite film annealed at 500 °C, with the inset showing a magnified view of an individual ZnO nanorod. Compared to the sample annealed at 300 °C, the film annealed at 500 °C exhibits a larger number of ZnO nanorods, which are also significantly longer, ranging from 10 μm to 30 μm in length. It is suggested that the higher annealing temperature significantly enhances the growth rate of the ZnO nanorods, facilitating them to reach the surface of the SiO_2_ substrates more rapidly.

### 3.3. EDS Analysis

**Figure 3:**
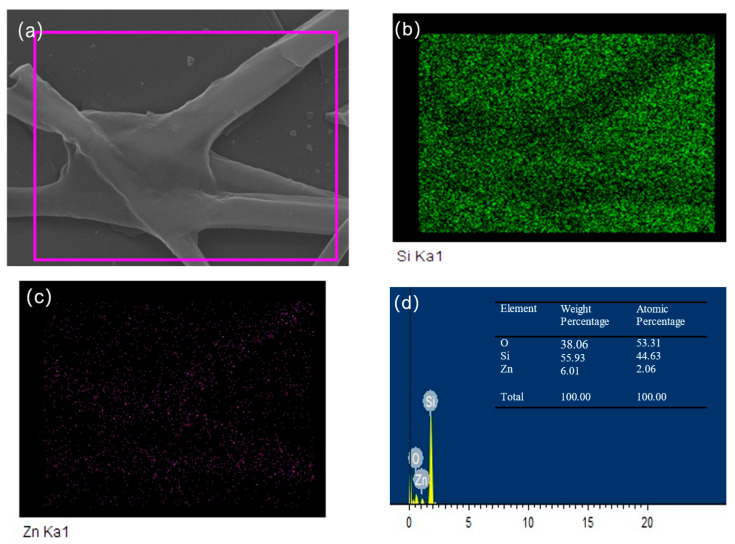
EDS mapping of ZnO-SiO_2_ films annealed at 500 °C: (**a**) the selected area; (**b**) Si elemental mapping; (**c**) Zn elemental mapping; (**d**) EDS spectrum.

Figure 3 shows the EDS mapping of ZnO-SiO_2_ films annealed at 500 °C. Figure 3a presents the selected area for EDS analysis, which contains rod-like structures. Figure 3b,c display the elemental mapping, with green points representing silicon (Si) atoms and blue points representing zinc (Zn) atoms. The green points are sparsely distributed in the regions corresponding to the rod-like structures observed in the SEM images, while the blue points are densely concentrated in these rod regions. The result further confirms that the rod-like structures are primarily composed of zinc (Zn) and oxygen (O) elements. Figure 3d presents the EDS spectrum, indicating that the mass percentage of zinc (Zn) in this region is approximately 6%. Combined with the XRD results, it can be inferred that these rod-like structures are ZnO nanorods.

### 3.4. Raman Spectroscopy

**Figure 4:**
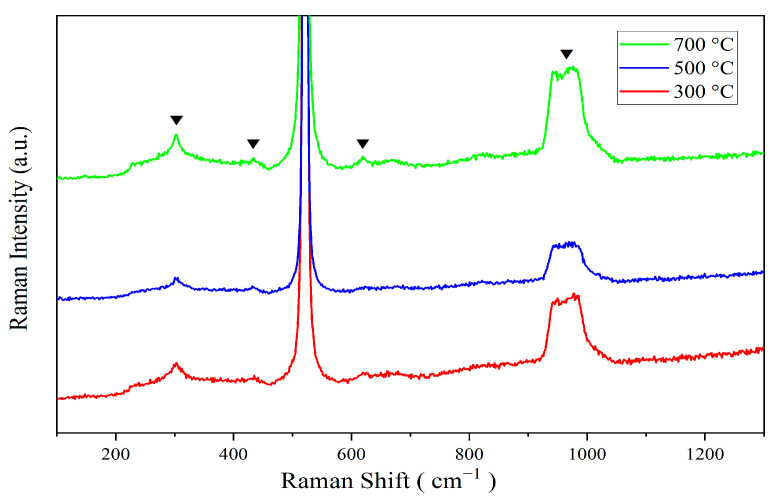
Raman patterns of annealed at 300 °C, 500 °C, and 700 °C.

Figure 4 presents the Raman spectra of ZnO-SiO_2_ thin films annealed at various temperatures. From Figure 4, distinct Raman shifts are observed at 303 cm^−1^, 432 cm^−1^, 617 cm^−1^, and a broad peak centered at about 960 cm^−1^. The peak at 303 cm^−1^ is generally attributed to the second-order phonon mode of the silicon substrate, specifically the second-order transverse acoustic (2TA) phonon mode in crystalline silicon [14]. Though relatively weak in standard Raman spectra, the peak at 303 cm^−1^ is observed in ZnO-SiO_2_ films with silicon substrates. The intensity of this Raman peak is relatively weaker at 500 °C annealing, possibly due to the interface stress between ZnO and SiO_2_ or structural changes in ZnO nanostructures, which suppress the vibrational mode of the substrate. In contrast, the intensity of the peak at 303 cm^−1^ is higher at 300 °C and 700 °C than that at 500 °C, where the crystal structure of the substrate is less affected. Particularly at 700 °C, the improved bonding quality between ZnO and SiO_2_ may enhance the signal of this vibrational mode.

The peak at 432 cm^−1^ corresponds to the bending vibration mode of Si-O-Si bonds within the SiO_2_, associated with bond angle variations, which are commonly observed in silicon oxide and amorphous SiO_2_ materials [14]. With increasing annealing temperature, the intensity of the peak at 432 cm^−1^ slightly increases. This may be attributed to the gradual ordering of the SiO_2_ structure as the annealing temperature rises, thereby enhancing the vibrational signal.

Additionally, a prominent peak around 520 cm^−1^ is attributed to the transverse optical (TO) phonon mode of the silicon substrate [15]. At 617 cm^−1^, a mode sometimes appears in ZnO, potentially related to local vibrational modes induced by defect states or lattice mismatches within the ZnO structure. The Raman mode near 617 cm^−1^ can also be attributed to the longitudinal optical (LO) mode of ZnO [16]. At an annealing temperature of 500 °C, the defect density in the ZnO nanostructure is relatively high, resulting in a weaker peak intensity. High-temperature annealing (700 °C) treatment results in reducing defects in ZnO and improving crystalline performance, which leads to a significant enhancement in the peak intensity.

Finally, the peak within the 960–980 cm^−1^ range is ascribed to surface phonon modes of the SiO_2_ substrates and/or ZnO nanoparticles, indicative of interactions at the ZnO-SiO_2_ interface or ZnO nanoparticle surfaces [17]. At 500 °C, the intensity of this peak is lowest, which may be attributed to significant interfacial stress or defects between ZnO and SiO_2_. As the annealing temperature increases, the interfacial stress is released, and the interaction between ZnO and SiO_2_ is enhanced, resulting in a significant increase in the peak intensity at 700 °C. In contrast, at 300 °C, the weaker bonding between ZnO and SiO_2_ leads to a relatively lower intensity of the surface phonon mode.

### 3.5. Photoluminescence (PL) Spectra

**Figure 5:**
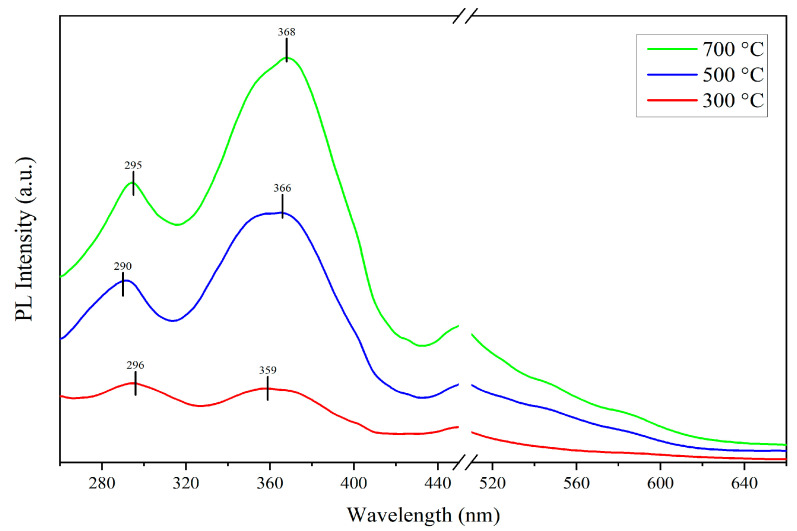
PL spectra of ZnO-SiO_2_ films annealed at 300 °C, 500 °C, and 700 °C.

The Figure 5 displays the photoluminescent (PL) spectra of samples prepared at different annealing temperatures with the excitation wavelength of 240 nm. All the samples exhibit two prominent PL peaks centered at around 296 nm and 360 nm. The ultraviolet emission at 296 nm is likely attributed to defects involving three-coordinated silicon atoms (O_3_≡Si-), where these defects capture holes and recombine with electrons to emit photons [18]. As the annealing temperature increases, the PL peak at 296 nm becomes sharper, which suggests that the higher annealing temperatures promote an increase in the density of O_3_≡Si- defects, thereby enhancing this ultraviolet emission.

The ultraviolet emission near 360 nm belongs to the near-band-edge (NBE) emission, which is characterized by a broader peak shape, indicating that this emission is influenced by multiple factors. The primary contribution to this ultraviolet emission originates from the donor level states formed by Zn–O–Si bonds at the ZnO–SiO_2_ interface [19]. Additionally, various transition processes within the ZnO nanorods [20] and defect states in the SiO_2_ substrates also significantly contribute to this ultraviolet emission. With increasing the annealing temperature, this emission band shows a redshift, and the peak shape becomes gradually sharper. The reason may be that the higher annealing temperature cause the donor level states of Zn–O–Si bonds to move towards lower energy and increase the density of Zn–O–Si bonds, thereby enhancing the dominant role of this emission [19].

In general, ZnO nanorods possess a large surface area-to-volume ratio and contain numerous dangling bonds, leading to significant visible light emission bands ranging from 500 to 650 nm [21]. However, the experimental results show a noticeable absence of emission bands around 560 nm in all the samples. This result is attributed to the fact that the ZnO nanorods grown on the SiO_2_ substrates form Zn–O–Si bonds, which reduce the density of dangling bonds on the surface of the ZnO nanorods, thereby suppressing the visible light emission bands. This reduction in surface trap-related visible emission increases the probability of ultraviolet emission via radiative recombination [19].

In summary, the variation in the PL spectra with annealing temperature indicates that the annealing process has a significant impact on defect formation and the electronic structure of the ZnO-SiO_2_ films. The higher annealing temperature promotes the formation of Zn–O–Si bonds, which plays a key role in the ultraviolet emission at 360 nm. Meanwhile, the temperature also increases the density of O_3_≡Si- defects, thereby enhancing the PL peak at 296 nm.

### 3.6. Photoelectric Properties Testing

**Figure 6:**
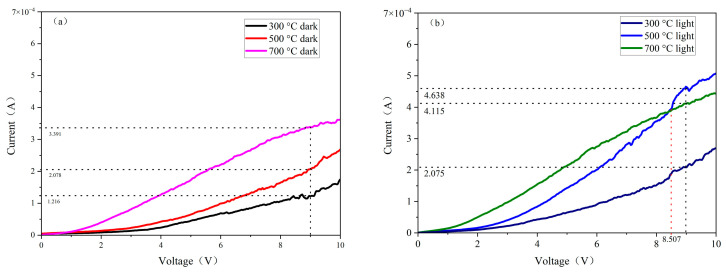
I-V curves of ZnO-SiO_2_ films at different light conditions: (**a**) dark (**b**) visible light.

To evaluate the photoresponse characteristics of the composite films, current–voltage (I–V) and current–time (I–T) tests were conducted. Figure 6 illustrates the I–V characteristics of ZnO-SiO_2_ films under dark and visible light conditions. As shown in Figure 6a,b, the measured I–V curves exhibit an overall nearly linear trend, particularly within the smaller voltage range, which indicates good Ohmic contact between the probes and the films [22]. This suggests that the films and the electrodes make good contact. As the annealing temperature increases, the conductivity of the films improves. Furthermore, under visible light illumination, the conductivity of all the samples shows different enhancements.

As shown in Figure 6b, under visible light illumination, the conductivity of the prepared films increases significantly, particularly for the film annealed at 500 °C. At the voltage of about 8.5 V, the photocurrent of this sample surpasses that of the film annealed at 700 °C.

Table 1 presents the current values of the films under various light conditions at a voltage of 9 V. The data in the table show that with the visible light condition, the photocurrents of the films annealed at 300 °C, 500 °C, and 700 °C increase by 70.64%, 126.86%, and 21.35%, respectively, compared to those in darkness. This indicates that the film annealed at 500 °C exhibits superior photoconductivity related to films annealed at other temperatures.

**Figure 7:**
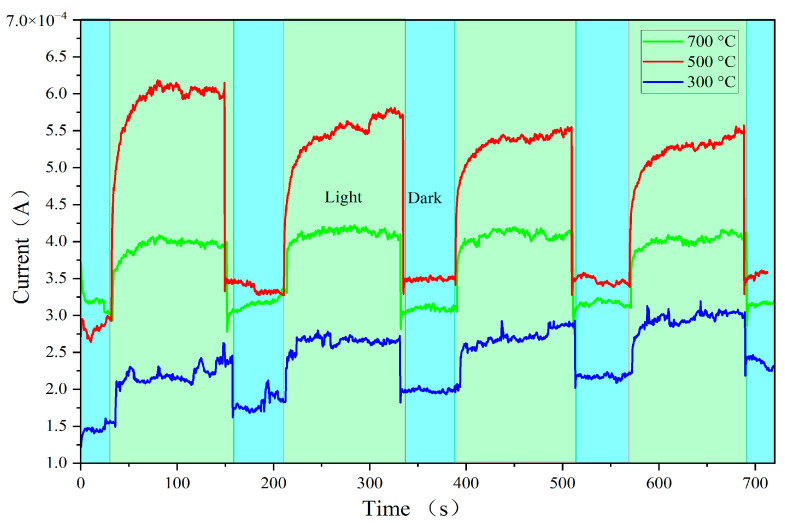
I-T curves of ZnO-SiO_2_ films at the voltage of 9 V.

Figure 7 presents the I–T curves of the prepared ZnO-SiO_2_ films at the voltage of 9 V. The repeatability of the films was tested by cyclically switching the visible light source on and off at equal time intervals. The on and off durations for visible light illumination were set to 120 s and 60 s, respectively. As shown in Figure 7, the current increases during the 120 s period of visible light illumination and then decreases to a steady state during the subsequent 60 s dark period. All the samples exhibit similar current trends of rise and fall over four cycles, indicating good reproducibility of the films. Among them, the film annealed at 500 °C shows the largest photocurrent jump and agrees with the I–V measurement results shown in Figure 6, indicating that 500 °C is the optimal annealing temperature for achieving the high photoconductivity of ZnO-SiO_2_ films.

**Figure 8:**
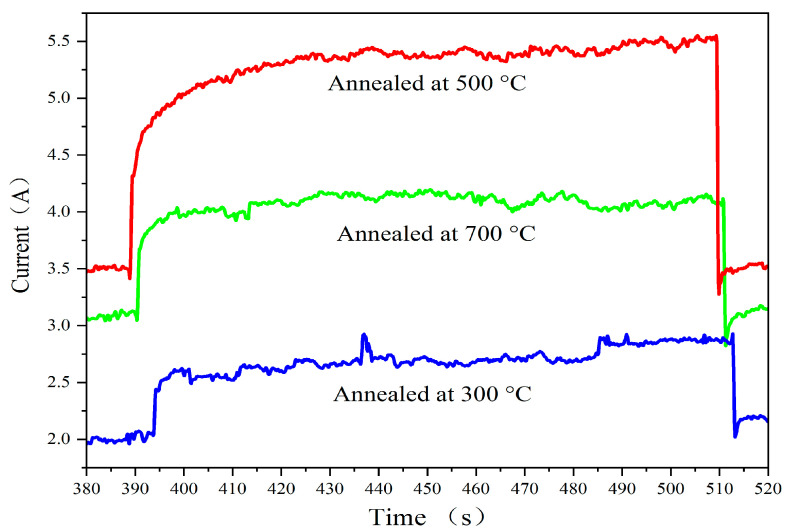
Response time measurements by visible light switched on and off at various annealing temperatures.

Figure 8 presents the response time measurements by visible light switched on and off at various annealing temperatures. The photoresponse time is a critical parameter for evaluating the speed at which a photoelectric device responds to light signals, involving both rise time (τR) and fall time (τD) [23]. The shorter the photoresponse time of a device, the greater its potential in practical photoelectric detection applications. The curves shown in Figure 8 are selected from Figure 7, ranging from 380 s to 520 s, which shows the time-resolved photocurrent of the films during the switching on and off of visible light illumination. At the voltage of 9 V, the rise time for films annealed at 300 °C, 500 °C, and 700 °C is approximately 1.5 s, 2.4 s, and 2.3 s, respectively, while the fall time is about 1.4 s, 2.7 s, and 2.1 s, respectively. The experimental results show that the best photoresponse time is approximately 2.9 s, indicating that the prepared films exhibit relatively fast visible light response properties under the given experimental conditions.

To further demonstrate the potential of the thin film as a photodetector, we calculated the responsivity (R) and detectivity (D*). R is given by [24]

R = (I_photo_ − I_dark_)/P·S, (1)

while D* is given by [25]

D* = R (S/2eI_dark_)^1/2^, (2)

where I_photo_ is the current under light illumination, I_dark_ is the current in the dark, P is the light power density, S is the active light area, and e is the electron charge. Based on equation 1, the responsivities of the films annealed at 300 °C, 500 °C, and 700 °C were calculated to be 29.4, 87.7, and 24.8 mA/W, respectively. Similarly, from equation 2, the detectivities of the films annealed at 300 °C, 500 °C, and 700 °C were calculated to be 4.7 × 10^10^, 1.1 × 10^11^, and 2.4 × 10^9^ Jones, respectively. Table 2 compares the performance parameters of the prepared thin films with those of photodetectors based on ZnO nanostructure materials, showing that our samples exhibit comparable detectivity with the previously reported work.

## 4. Discussion

**Figure 9:**
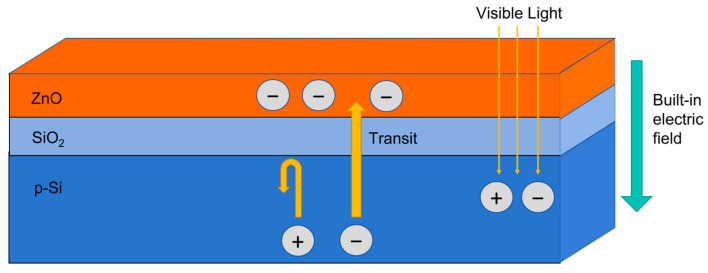
A schematic diagram of carrier transport in the ZnO-SiO_2_ thin film on a Si substrate under visible light illumination.

Figure 9 is a schematic diagram of carrier transport in the ZnO-SiO_2_ thin film on a Si substrate under visible light illumination. When visible light irradiates the ZnO-SiO_2_ thin films, a portion of the light transmits through the film and is absorbed by the Si substrate, generating electron–hole pairs. Under the influence of the built-in electric field, the photogenerated electrons pass through the SiO_2_ into the ZnO [34], while the SiO_2_ effectively blocks the holes [35]. This blocking action reduces the carrier recombination rate within ZnO, resulting in an increased carrier density in the film and a reduction in the resistance, rapidly promoting the photocurrent. When the sample transits from a light-illuminated environment to a dark environment, additional photogenerated electrons are no longer produced, and the existing photogenerated electrons are rapidly depleted by the SiO_2_, causing the film resistance to quickly return to its initial value.

The sample annealed at 500 °C exhibits better optoelectronic performance, likely because at this temperature, highly ordered ZnO nanorods with good crystallinity can be grown, while also achieving an ideal ZnO-SiO_2_ interface structure. At this point, the thickness of the SiO_2_ layer is within the ideal range: thick enough to act as an effective hole barrier to suppress recombination, yet not too thick to hinder electron injection. This balance is crucial for maximizing the carrier transport efficiency and ensuring a good photoelectric response.

In contrast, at 300 °C, the thinner SiO_2_ layer is not effective at blocking holes, reducing the carrier separation efficiency and resulting in a lower photocurrent. At 700 °C, although the crystallinity of the ZnO nanorods improves, the excessively thick SiO_2_ layer introduces resistance to electron transport, weakening the injection efficiency of photogenerated electrons into the ZnO nanorods. Therefore, the appropriate annealing condition can achieve the optimal balance between injection efficiency and recombination suppression, ultimately leading to the best photoelectric performance.

## 5. Conclusions

In this study, ZnO-SiO_2_ composite films were successfully fabricated on silicon substrates using a simple sol–gel method combined with spin-coating. XRD analysis confirmed the hexagonal wurtzite structure of ZnO, and diffraction peak shifts were observed, attributed to compressive strain. SEM and EDS spectral analysis further verified the presence of ZnO nanorods and their strong integration with the SiO_2_ matrix. The photoluminescence (PL) spectra demonstrated that high-temperature annealing significantly enhanced the ultraviolet emission while suppressing the visible light emission.

In terms of the photoelectric performance, the annealing temperature had a pronounced impact on the film’s conductivity and photoresponse. The film annealed at 500 °C exhibited excellent photoconductivity and photoresponse, further exhibiting the potential application of ZnO-SiO_2_ composite films in optoelectronic devices. Overall, the ZnO-SiO_2_ films prepared in this study have excellent photoelectric properties, offering broad application prospects in the field of optoelectronic devices.

## Figures and Tables

**Table 1:** The current values of the films with various light conditions at a voltage of 9 V.

Annealing Temperature (°C)	Light Current (10^−4^ A)	Dark Current (10^−4^ A)	Increase in Ratio (%)
300	2.075	1.216	70.64
500	4.638	2.078	126.86
700	4.115	3.391	21.35

**Table 2:** Performance parameters of device based on ZnO nanostructure materials.

Device	Wavelength (nm)	Voltage (V)	R_max_ (mA/W)	D*_max_ (Jones)	Ref.
ZnO NWs	360	1	390	1.9 × 10^8^	[26]
ZnO NWs	310	3	—	—	[27]
ZnO/Si	365	−2	340	2.11 × 10^10^	[28]
ZnO/CuO/GaN	365	0	1.44	5.9 × 10^9^	[29]
AZO/n-Si	365	−2	150	—	[30]
In-doped ZnO NRs	UV	1	2500	1.44 × 10^11^	[31]
ZnO/CuO	365/405/515	0	1.2 × 10^−3^	1.8 × 10^7^	[3]
p-CuSCN/n-ZnO NRs	365	−3	22.5	—	[32]
Electrospun ZnO NWs/PbS QDs	350	10	51	3.4 × 10^8^	[33]
ZnO-SiO2	VIS	9	87.7	1.1 × 10^11^	This work

## Data Availability

Data are contained within the article.
